# ACSS2 involved in acetyl‐CoA synthesis regulates skeletal muscle function

**DOI:** 10.1002/1873-3468.70152

**Published:** 2025-09-12

**Authors:** Mekala Gunasekaran, Gloriana Campos, Natalya M. Wells, Khanhlinh Lambuu, Isabelle Draper, Christina A. Pacak, Peter B. Kang

**Affiliations:** ^1^ Greg Marzolf Jr. Muscular Dystrophy Center and Department of Neurology University of Minnesota Medical School Minneapolis MN USA; ^2^ Molecular Cardiology Research Institute Tufts Medical Center Boston MA USA; ^3^ Institute for Translational Neuroscience University of Minnesota Minneapolis MN USA

**Keywords:** ACSS2, cholesterol metabolism, muscle development

## Abstract

Acyl‐coenzyme A synthetase short‐chain family member‐2 (ACSS2) catalyzes the conversion of acetate to acetyl‐CoA, regulating cholesterol metabolism. Given the discovery of a muscular dystrophy associated with 3‐hydroxy‐3‐methyl‐glutaryl‐coenzyme A reductase (HMGCR), a key enzyme in cholesterol synthesis, we studied *Acss2* in mice and the orthologous gene *AcCoA* in flies. Skeletal muscle from *Acss2* ^−/−^ mice showed atrophic fibers, lipid accumulation, and depleted NADH levels, while myoblasts from these mice displayed precocious differentiation. Exercise induced fatigue in the *Acss2* ^−/−^ mice, which was accentuated by inhibition of ATP‐citrate lyase (ACLY) activity. *AcCoA* knockdown yielded reduced body sizes and locomotor defects in *Drosophila*. ACSS2 is vital for skeletal muscle function and merits study as a potential factor in muscle diseases related to cholesterol metabolism.

**Impact statement**

ACSS2 catalyzes the conversion of acetate to acetyl‐CoA, regulating cholesterol metabolism. Given the increasingly apparent links between cholesterol metabolism and skeletal muscle function, we investigated ACSS2 deficiency in mouse and fly models. We identified defects in muscle morphology, muscle metabolism, and motor function. ACSS2 is vital for skeletal muscle.

## Graphical abstract

The enzyme acyl‐coenzyme A synthetase short‐chain family member‐2 (ACSS2) catalyzes the conversion of acetate to acetyl‐CoA, but its function in skeletal muscle is unclear. We studied ACSS2 deficiency in mouse and fly models. Skeletal muscle from the mouse model showed atrophic fibers, excess lipid, and depleted NADH. The fly model showed locomotor defects. ACSS2 is important for skeletal muscle function.

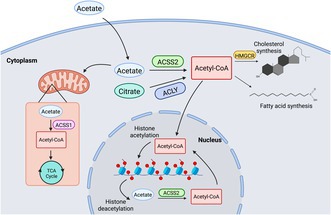

## Abbreviations

**ACLY**, ATP‐citrate lyase

**ACSS2**, Acyl‐coenzyme A synthetase short‐chain family member‐2

**GGPS1**, geranylgeranyl diphosphate synthase 1

**HAT**, histone acetyltransferase

**HMGCR**, 3‐hydroxy‐3‐methyl‐glutaryl‐coenzyme A reductase

**HMGCS1**, 3‐hydroxy‐3‐methylglutaryl‐CoA synthase 1

**PDH**, pyruvate dehydrogenase

**TCA**, tricarboxylic acid

**WT**, wild type

**Fig. 1:**
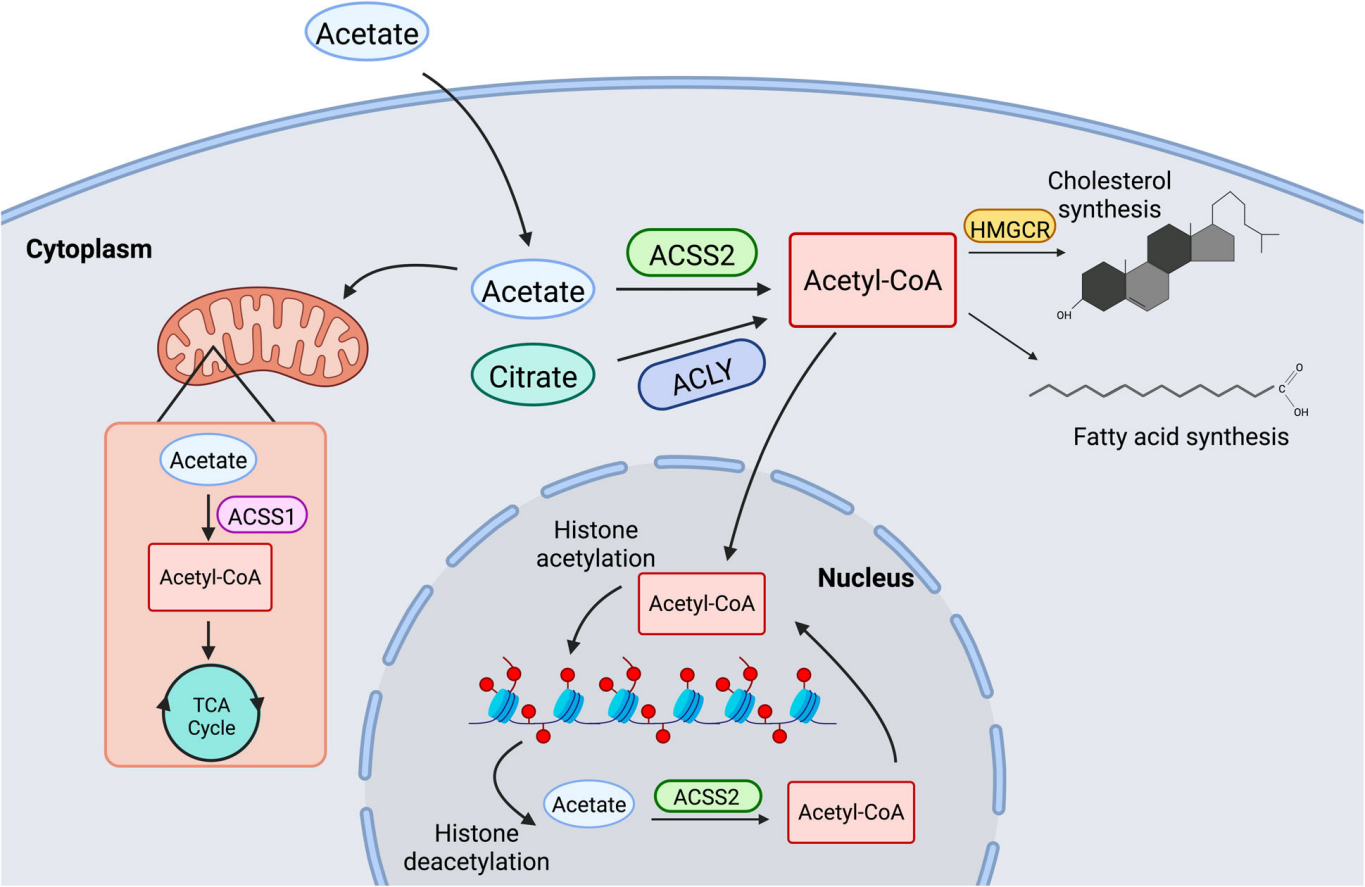
Illustration of the molecular pathway of ACSS2 and ACLY mediated acetyl‐CoA generation. Acetyl‐CoA serves as the central regulator of two major downstream metabolic pathways of fatty acid and cholesterol synthesis. The diagram was generated using BioRender under a license permitting its use in publications.

The *ACSS2* gene encodes acyl‐coenzyme A synthetase short‐chain family member‐2 (ACSS2), an enzyme that catalyzes the ATP‐dependent conversion of free acetate to acetyl‐coenzyme A (acetyl‐CoA) and AMP generation in the cytosolic compartment (Fig. 1). In the nucleus, ACSS2 contributes to the histone acetylation process that recaptures acetate from histone deacetylation and forms acetyl‐CoA, which is then used as a substrate for histone acetyltransferases (HATs) [1]. There are three known isoforms of human *ACSS* genes: *ACSS1*, *ACSS2*, and *ACSS3*, with protein products known under the same abbreviations. ACSS1 is known to be localized to mitochondria and drives the tricarboxylic acid (TCA) cycle. ACSS1 regulates fasting‐induced sarcopenia through mitochondrial dysfunction in mice [2]. Deficiency of ACSS3, found in brown adipose tissues, is linked to idiopathic pulmonary fibrosis (IPF) [3] and increased fat mass *in vivo* [4]. ACSS2 localizes to the cytosol and nucleus, regulating lipid and fatty acid biosynthesis. The Sirtuin (SIRT) family of nicotine adenine dinucleotide (+)‐dependent histone deacetylases is involved in biological processes such as inflammation, apoptosis, metabolism, and oxidative stress [5]. ACSS2 is activated by Sirtuin1 (SIRT1) mediated deacetylation in the nucleus and cytoplasm, while Sirtuin3 (SIRT3) activates ACSS3 in the mitochondrial matrix [6, 7].

ATP‐citrate lyase (ACLY) is an enzyme similar to a Sirtuin that also catalyzes the conversion of citrate to acetyl‐CoA [8, 9]. ACSS2 and ACLY both contribute to the cytosolic acetyl‐CoA pool, which is the primary substrate for the activation of two major metabolic pathways: the fatty acid synthesis pathway and the mevalonate cholesterol synthesis pathway [10]. Other sources of acetyl‐CoA are available, including the pyruvate‐mediated conversion of glucose to acetyl‐CoA using pyruvate dehydrogenase (PDH) in mitochondria, which then contributes to the citrate pool [11]. Several components of the mevalonate pathway have now been linked to musculoskeletal disorders, including muscular dystrophy: 3‐hydroxy‐3‐methyl‐glutaryl‐coenzyme A reductase (*HMGCR*, limb‐girdle muscular dystrophy) [12, 13, 14], geranylgeranyl diphosphate synthase 1 (*GGPS1*, muscular dystrophy with hearing loss/ovarian insufficiency syndrome) [15], and 3‐hydroxy‐3‐methylglutaryl‐CoA synthase 1 (*HMGCS1*, rigid spine syndrome) [16]. There is a link between the mevalonate pathway and the common cholesterol‐lowering statin drugs, which inhibit HMGCR and, curiously, are well known to cause muscle side effects [17].

Metabolic signaling pathways displayed differing patterns of alterations in the liver, brain, and adipose tissue of mice [17]. *Acss2* knockout mice fed a high‐fat diet lost body weight and experienced less hepatic steatosis due to a reduction of dietary lipid absorption in the intestine [18]. Utilization of triglycerides in the liver was altered due to the differential expression of lipid transporters and fatty acid oxidation genes [18]. These data suggest that ACSS2 regulates fat storage and metabolism.

Given the metabolic links between ACSS2 and HMGCR, along with the association of HMGCR with muscular dystrophy, we hypothesized that ACSS2 also regulates muscle development and function and tested this hypothesis in a mouse knockout model and a fly knockdown model.

## Materials and methods

### *Acss2* ^−/−^ mouse breeding

All vertebrate animal work was conducted under a protocol approved by the Institutional Animal Care and Use Committee (IACUC) at the University of Minnesota. Heterozygous *Acss2* ^+/−^ mice were obtained from Jackson Laboratory (Bar Harbor, ME, USA; JAX stock #033565). All mice were maintained in an Association for Assessment and Accreditation of Laboratory Animal Care International (AAALAC) accredited Research Animal Resources (RAR) facility at the University of Minnesota. The RAR conditions were 25 °C temperature and 50% humidity in a 14‐h light and 10‐h dark cycle, with the mice being fed normal chow and receiving water *ad libitum*. Veterinary care was provided by RAR staff for all animals throughout the study. We bred the heterozygous mice to generate homozygous *Acss2* ^−/−^ mice. Homozygosity was confirmed via PCR‐based genotyping using primers recommended by Jackson Laboratory. *Acss2* ^+/+^ mice from these crosses were used as wild‐type (WT) controls. The experiments ended at 11 weeks of age, with both male and female mice being euthanized at that time point.

### Body and tissue weight measurements

The body weights of *Acss2* ^−/−^ and WT mice were measured weekly from 3 to 100 weeks of age. Lengths and tissue weights were measured during necropsy at 11 weeks of age.

### Barium chloride muscle injury

*Acss2* ^−/−^ and WT 8‐week‐old mice received intramuscular injections with a 1.2% barium chloride solution in the tibialis anterior (TA) muscle on one side; the contralateral TA muscle was injected with sterile saline (V1 510 224, Vet One, Bimeda‐MTC Animal Health Inc., Cambridge, Ontario, Canada). The mice were euthanized at 1, 5, and 12 days after injury for tissue collection.

### Exercise and locomotor activity assessments before and after fasting

*Acss2* ^−/−^ and WT mice exercised on a treadmill for 5 min at 8 cm·s^−1^ (5 m·min^−1^), followed by 10 min at 16 cm·s^−1^ (10 m·min^−1^) at a 15‐degree incline, followed by locomotor activity measurements via Actitrack for 5 min. The mice were then fasted for 16 h (with free access to water), followed by tail vein phlebotomy to measure serum glucose levels (AlphaTrak Blood Glucose Monitoring System, Zoetis Inc., Parsippany, NJ, USA). The exercise and locomotor activity measurements were repeated after fasting.

### ACLY inhibitor administration

An ACLY inhibitor (BMS303141, MedChemExpress, Princeton, NJ, USA) was administered at 10 mg·kg^−1^ in sterile saline (V1 510224, Vet One) to 8‐week‐old *Acss2* ^−/−^ and WT mice by oral gavage once daily for 7 days. The exercise and locomotor activity assessments were repeated before and after fasting at the end of the ACLY inhibitor treatment period.

### Social behavior experiments

Social preferences and recognition were quantified in 10‐week‐old *Acss2* ^−/−^ and WT mice using a 3‐Chamber Social Interaction Assay (ANY‐maze, Stoelting, Wood Dale, IL, USA). A single experimental mouse was placed in the middle chamber for 5 min during the Habituation Stage. In the Sociability Test, a stranger mouse (Stranger 1) was placed in a cage in a side chamber, while the experimental mouse could move freely throughout the three chambers for 10 min. In the Social Novelty Test, a second stranger mouse (Stranger 2) was placed in a cage in the other side chamber, while the experimental mouse could again move freely throughout the three chambers for 10 min.

### Primary mouse myoblast isolation

One‐ to five‐day‐old mouse pups were genotyped via toe snips. The pups were euthanized and skeletal muscles from the hind limbs were extracted using a skeletal muscle isolation kit (Miltenyi Biotec, Bergisch Gladbach, Germany). The cells were then seeded on a Matrigel‐coated plate for 2 days in F‐10 nutrient mix (Gibco, Thermo Fisher Scientific, Waltham, MA, USA) + bFGF + 1% Penicillin–Streptomycin (Pen‐Strep) medium, and later the pre‐plating step was carried out in F‐10 nutrient mix (Gibco) + DMEM + 10% FBS + bFGF + 1% Pen‐Strep medium to remove fibroblasts and enhance the growth of myoblasts. The pre‐plating step was repeated until a pure primary myoblast population was obtained.

### Primary mouse myoblast differentiation

Primary mouse myoblasts were seeded on a Matrigel‐coated plate and cultured in growth medium containing F‐10 nutrient mix (Gibco) + DMEM + 10% FBS + bFGF + 1% Pen‐Strep. At 60% confluence, the medium was changed to differentiation medium containing 5% horse serum (Gibco). After 4 days of differentiation with daily media changes, the cells were fixed and permeabilized using 4% PFA for 15 min. After permeabilizing the cells with Triton X, the cells were stained with anti‐MyHc antibody (DSHB‐MF‐20) for 1 h followed by Alexa‐568 mouse secondary antibody for 1 h. The cells were then stained with DAPI for 5 min and mounted onto glass slides using DAPI gold antifade mounting medium. The slides were imaged using an epifluorescent microscope (Leica Thunder, Wetzlar, Germany). The myotube fusion index was calculated by counting the total number of nuclei in the myotubes divided by the total number of nuclei within MHC‐positive myotubes and divided by the total number of nuclei in the field of view using ImageJ (National Institutes of Health, Bethesda, MD, USA).

### RNA isolation and qPCR

RNA isolation from primary mouse myoblasts was performed using an RNA isolation kit (Zymo Research, Irvine, CA, USA), followed by reverse transcription using a High‐Capacity RNA to cDNA Kit (Applied Biosystems, Foster City, CA, USA). RT‐qPCR‐based gene expression analysis was conducted using the Taqman Fast Advanced Master Mix in a QuantStudio 3 Real‐Time PCR System (Applied Biosystems). We used Taqman probes to measure expression levels of *MyoD* (Mm00440387_m1, Thermo Fisher, Waltham, MA, USA), *MyoG* (Mm00446194_m1, Thermo Fisher) and *Gapdh* (Mm99999915_g1, Thermo Fisher).

### Hematoxylin and eosin (H&E) staining

After placement on glass slides, 8 μm thick mouse quadriceps and TA sections were air dried for 20 min, then stained in Harris hematoxylin (HHS16‐500ML, Sigma‐Aldrich, St. Louis, MO, USA) for 2 min. The slides were rinsed with tap water for 1 min, then dipped in 80% alcohol for 30 s. The slides were placed in Alcoholic‐Eosin‐Y (3801615, Leica, Wetzlar, Germany) for 30 s, dehydrated in four changes of 100% alcohol, and cleared in three changes of xylene. Permount (SP15‐100, Fisher Chemical, Thermo Fisher Scientific, Waltham, MA, USA) was used to mount and coverslip the slides.

### NADH staining

Mouse quadriceps sections on glass slides were incubated in NADH‐NBT solution (nicotinamide adenine dinucleotide, reduced (NADH)) (Sigma N8129) and Nitro‐Blue Tetrazolium (Sigma N6876) for 30 min at 37 °C. The sections were then washed with three exchanges of tap or deionized H_2_O. The unbound NBT was removed from the sections with three exchanges each of 30%, 60%, and 90% acetone solutions. The sections were left in 90% acetone until a faint purple and cloudy appearance was seen over the sections. Finally, the slides were rinsed several times with deionized H_2_O and then mounted with an aqueous mounting medium containing glycerol.

### Periodic acid Schiff (PAS) staining

Mouse quadriceps sections on glass slides were fixed in methanol for 5 min. They were washed with dH_2_O and then with fresh 0.5% periodic acid for 10 min. The slides were rinsed with dH_2_O and stained with Schiff Reagent (6073‐71, Sigma‐Aldrich) for 10 min. They were washed with three exchanges of dH_2_O and dehydrated sequentially in 50%, 70%, 80%, 95%, and 100% ethanol. Three washes of xylene were performed to clear the tissues, and Permount (SP15‐100, Fisher Chemical) was used to mount the slides.

### Oil red O (ORO) staining

Mouse quadriceps sections on glass slides were air dried for 30 min. In the meantime, a working ORO solution was made by mixing 30 mL of stock solution (O1391‐250ML, MilliporeSigma, Burlington, MA, USA) with 20 mL of dH_2_O. The solution was left to stand for 10 min, then filtered with filter paper. The slides were fixed in 10% neutral buffered formalin for 10 min. They were then dipped in fresh 60% isopropanol and stained with the ORO working solution for 15 min. The sections were dipped in 60% isopropanol, dipped in dH_2_O, and counterstained with Mayer's Hematoxylin (MHS16‐500ML, Sigma‐Aldrich) for 10 min. Lastly, slides were dipped in dH_2_O ten times and cover slipped with glycerol gelatin mounting media (GG1‐15ML, Sigma‐Aldrich).

### Fiber typing via immunofluorescence

Mouse soleus and EDL sections on glass slides were air dried for 30 min and rinsed in 1× TBS in 0.1% Tween20 (TBST, 9005‐64‐5, Fisher BioReagents, Thermo Fisher Scientific, Waltham, MA, USA). The tissues were blocked for 30 min with 2.5% goat serum in TBST (005‐000‐121, Jackson ImmunoResearch Laboratories, West Grove, PA, USA). After blocking, the slides were incubated for 2 h at room temperature in one of the following primary antibodies: Type 1 Slow Myosin (Developmental Studies Hybridoma Bank (DSHB), Iowa City, IA, USA, AF.840; mouse IgGM, 1 : 100), Type 2a Fast Myosin (DSHB, SC‐71; mouse IgG1, 1 : 200), and Laminin 2a (ab11576, Abcam, Cambridge, UK, rat IgG, 1 : 200). Each slide was rinsed with TBST for 5 min 3 times, incubated for 1 h at room temperature in one of the following secondary antibodies: 594 Goat anti‐mouse IgGM (AlexaFluor A21145, 1 : 400), 488 Goat anti‐mouse IgG1 (AlexaFluor A21121, 1 : 400), and 568 Goat anti‐rat IgG (H + L) (AlexaFluor A11077, 1 : 400). The slides were washed with TBST for 5 min 3 times and rinsed with dH_2_O. Fluoromount Aqueous Mounting Medium (F4680‐25ML, Sigma‐Aldrich) was used to mount and coverslip the slides, then visualized using an epifluorescent microscope (Leica Thunder).

### Immunoblotting

Mice quadriceps muscles were lysed using RIPA buffer constituted with PMSF and sodium orthovanadate. The samples were lysed at 4 °C for 30 min with rotation. They were centrifuged to pellet debris at 15 000 g for 20 min at 4 °C. The supernatant was then subjected to a detergent compatible (DC) assay to measure the total protein concentration of each sample. Protein lysates were mixed with 4× LDS buffer and boiled at 95 °C for 5 min. Samples were resolved in a 4–20% SDS/PAGE gel (Bio‐Rad Laboratories, Hercules, CA, USA) and transferred onto a nitrocellulose membrane using the Trans‐Blot Turbo System (Bio‐Rad Laboratories). The blots were blocked for 1 h in 5% milk in TBST and then incubated with primary antibodies against Rb‐ACLY (Proteintech Group, Rosemont, IL, USA, 15 421‐1‐AP) and Rb‐GAPDH (Cell Signaling Technology, Danvers, MA, USA, 14C10) at 1 : 1000 overnight at 4 °C. The next day, the blots were washed with 1× TBST for 5 min three times. Then, the blots were incubated with the corresponding rabbit secondary HRP (Abcam‐ab6721) at 1 : 2000 concentration for 1 h at room temperature. The blots were again washed in 1× TBST for 5 min three times. Then the blots were developed using *ECL* chemiluminescence solution and visualized with the Jess Protein Simple System (Bio‐Techne, Minneapolis, MN, USA).

### AcCoA knockdown *Drosophila*

RNAi mediated knockdown of *AcCoA* (the fruit fly ortholog of *ACSS2*) was generated in flies at 29 °C using Bloomington stock #41917, Genotype: (y[1]sc[*]v[1]sev [21];P{y[+t7.7]v[+t1.8] = TRiP.HMS02314}attP2). Drivers specific to various tissue targets of interest were used. Viability, phenotypic features, and locomotor ability were analyzed as previously described [19].

## Results

### Characterization of *Acss2* ^−/−^ mice

**Fig. 2:**
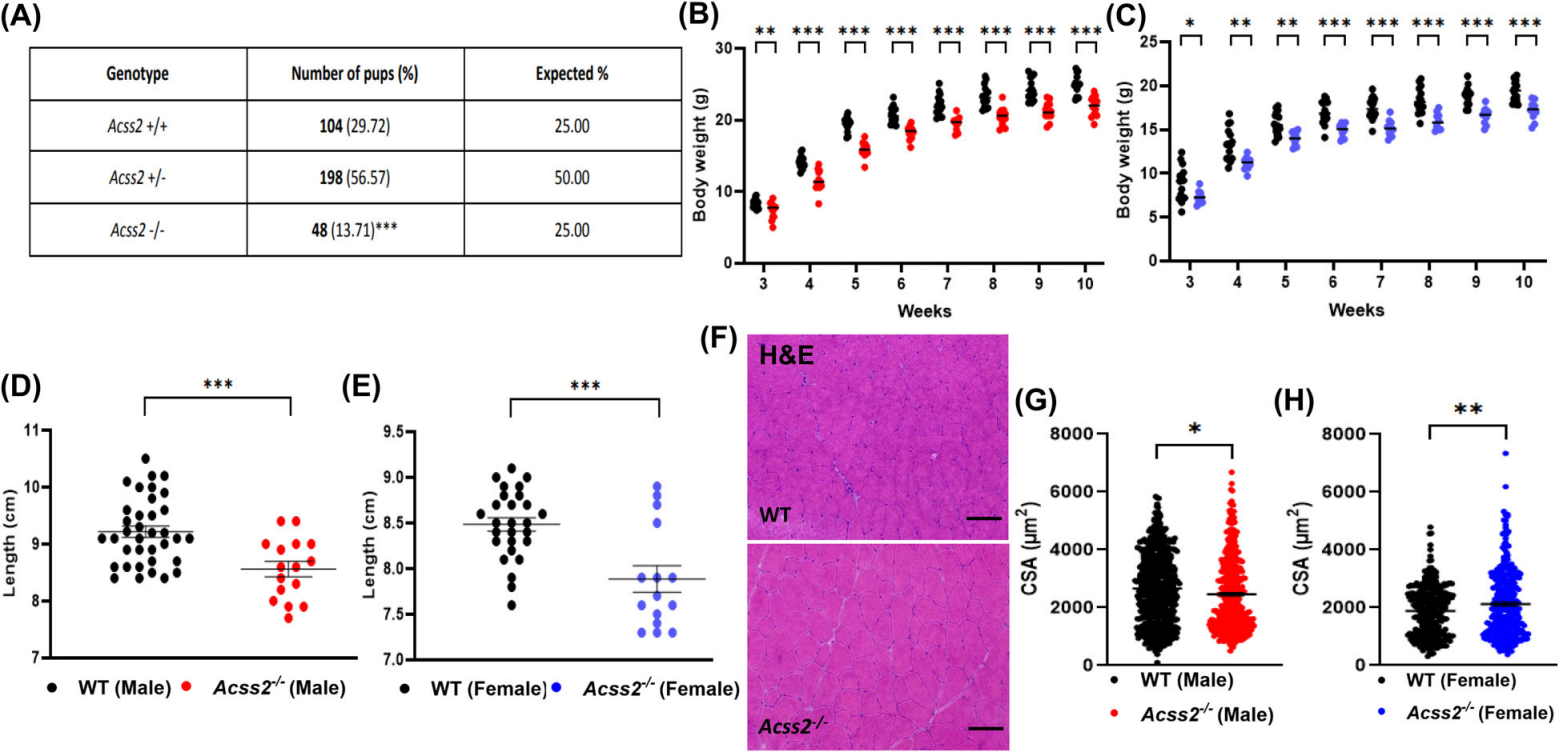
General phenotypic and histological features of *Acss2* ^−/−^ mice. (A) The observed proportion of *Acss2* ^−/−^ homozygous mice is significantly lower than expected across 61 litters. A chi‐square test shows ***, *P* < 0.001. (B) Male and (C) female *Acss2* ^−/−^ mice have lower body weights than wild‐type (WT) mice. The graphs show body weight measurements at 3 to 10 weeks, representing *n* = 15 (male WT), *n* = 12 (male *Acss2* ^−/−^), *n* = 16 (female WT), and *n* = 10 (female *Acss2* ^−/−^). (D) Male and (E) female *Acss2* ^−/−^ mice are shorter than WT mice. The data represent *n* = 36 (male WT), *n* = 16 (male *Acss2* ^−/−^), *n* = 27 (female WT), and *n* = 15 (female *Acss2* ^−/−^). (F) Representative microscopic images of hematoxylin and eosin (H&E) staining in WT and *Acss2* ^−/−^ mouse quadriceps. Scale bar, 50 μm. H&E staining showed varying muscle fiber sizes for *Acss2* ^−/−^ mice. (G and H) Histological sections of mouse quadriceps from *Acss2* ^−/−^ and WT mice were stained with H&E and cross‐sectional areas (CSA) of 100 muscle fibers from each quadriceps were quantified using imagej. An unpaired *t*‐test shows *, *P* < 0.05; **, *P* < 0.01; and ***, *P* < 0.001. Error bars show standard error of the mean (SEM).

**Fig. 3:**
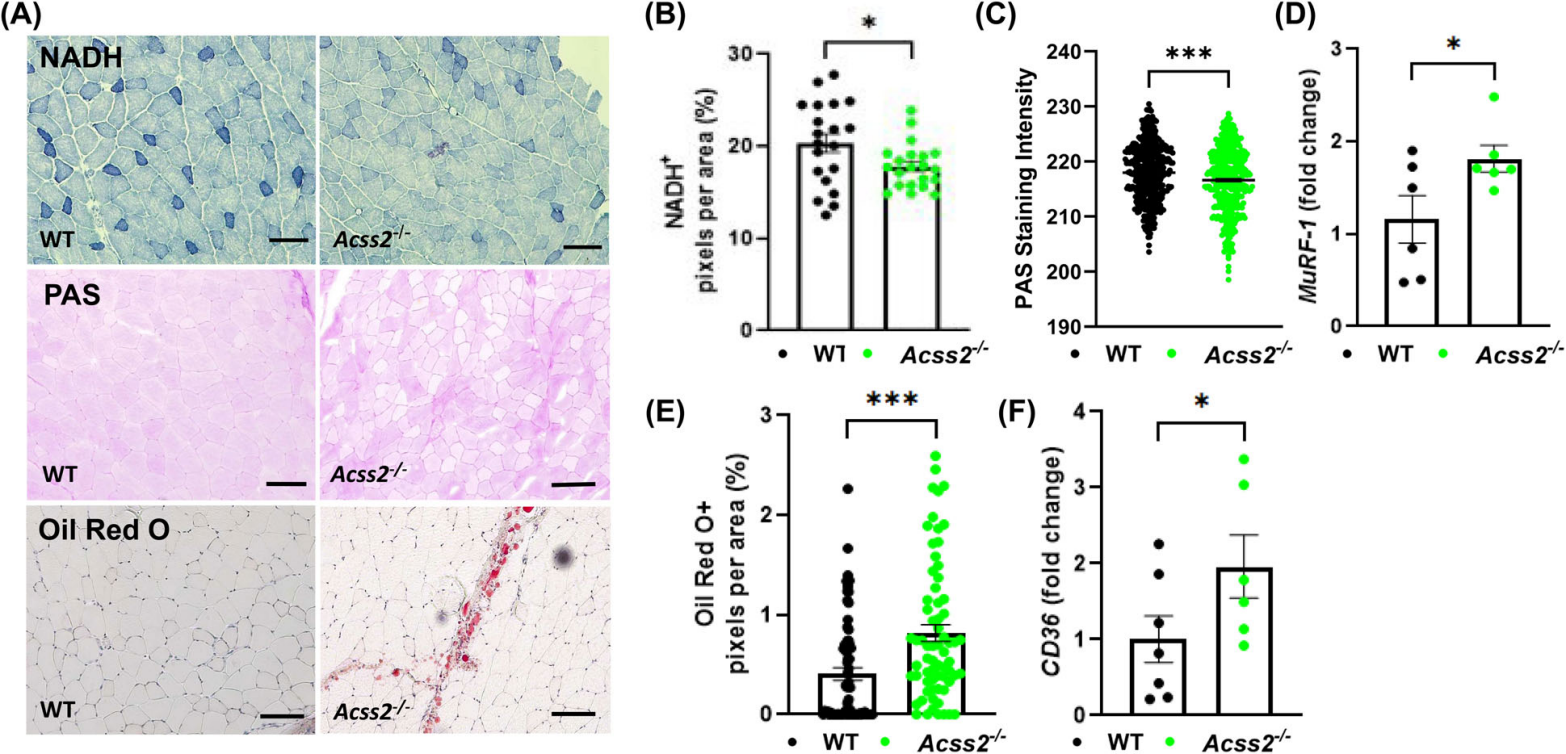
Muscle features of *Acss2* ^−/−^ mice. (A) Representative microscopic images of NADH, PAS, and Oil Red O staining in *Acss2* ^−/−^ and WT mice quadriceps. Scale bar, 50 μm. (B) NADH staining showed decreased abundance of fibers with positive NADH activity (dark blue fibers). (C) PAS staining of quadriceps showed increased glycogen in the muscle fibers of *Acss2* ^−/−^ mice, where staining intensity is based on a gray scale (255 = white, 0 = black). (D) Gene expression levels of the muscle atrophy marker *MuRF‐1* were measured in RNA obtained from quadriceps muscle specimens using qPCR. *MuRF‐1* was upregulated in *Acss2* ^−/−^ mouse muscle versus WT. (E) The percentages of pixels positive for Oil Red O in 10 fields of view covering entire muscle sections were captured and quantified using imagej. Each value represents the percent of pixels that are positive staining per muscle section, with one muscle section quantified per mouse. (F) qPCR analysis showed increased expression of *CD36* in *Acss2* ^−/−^ mouse quadriceps compared to WT. An unpaired *t*‐test shows *, *P* < 0.05 and ***, *P* < 0.001. Error bars show standard error of the mean (SEM).

We observed fewer *Acss2* ^−/−^ homozygous mice than would be expected from our crosses of heterozygous *Acss2* ^+/−^ mice, and the homozygous mice were infertile (Fig. 2A). *Acss2* ^−/−^ mice of both sexes had lower body weight than age‐matched wild‐type (WT) mice when weighed from 3 to 10 weeks of age (Fig. 2B,C). *Acss2* ^−/−^ mice of both sexes were shorter than WT mice (Fig. 2D,E). In contrast, the weights of different skeletal muscle tissues were similar between strains for both sexes (Fig. S1). The fiber typing analysis showed no significant differences in the soleus and EDL muscles (Fig. S2). H&E staining showed mild differences in muscle fiber size between the *Acss2* ^−/−^ and WT mice (Fig. 2F–H). NADH staining (Fig. 3A,B) was reduced, and Periodic acid Schiff staining (PAS) (Fig. 3A,C) was more variable but overall increased in *Acss2* ^−/−^ muscle compared to WT muscle. On qPCR, expression levels of the muscle atrophy marker *MuRF‐1* were increased in *Acss2* ^−/−^ compared to WT mice (Fig. 3D). Oil Red O (ORO) staining was increased in the extracellular matrix (Fig. 3A,E), while the expression of *CD36*, a fatty acid reporter gene, was upregulated in *Acss2* ^−/−^ muscles compared to WT muscles (Fig. 3F).

### Muscle regeneration in *Acss2* ^−/−^ mice

**Fig. 4:**
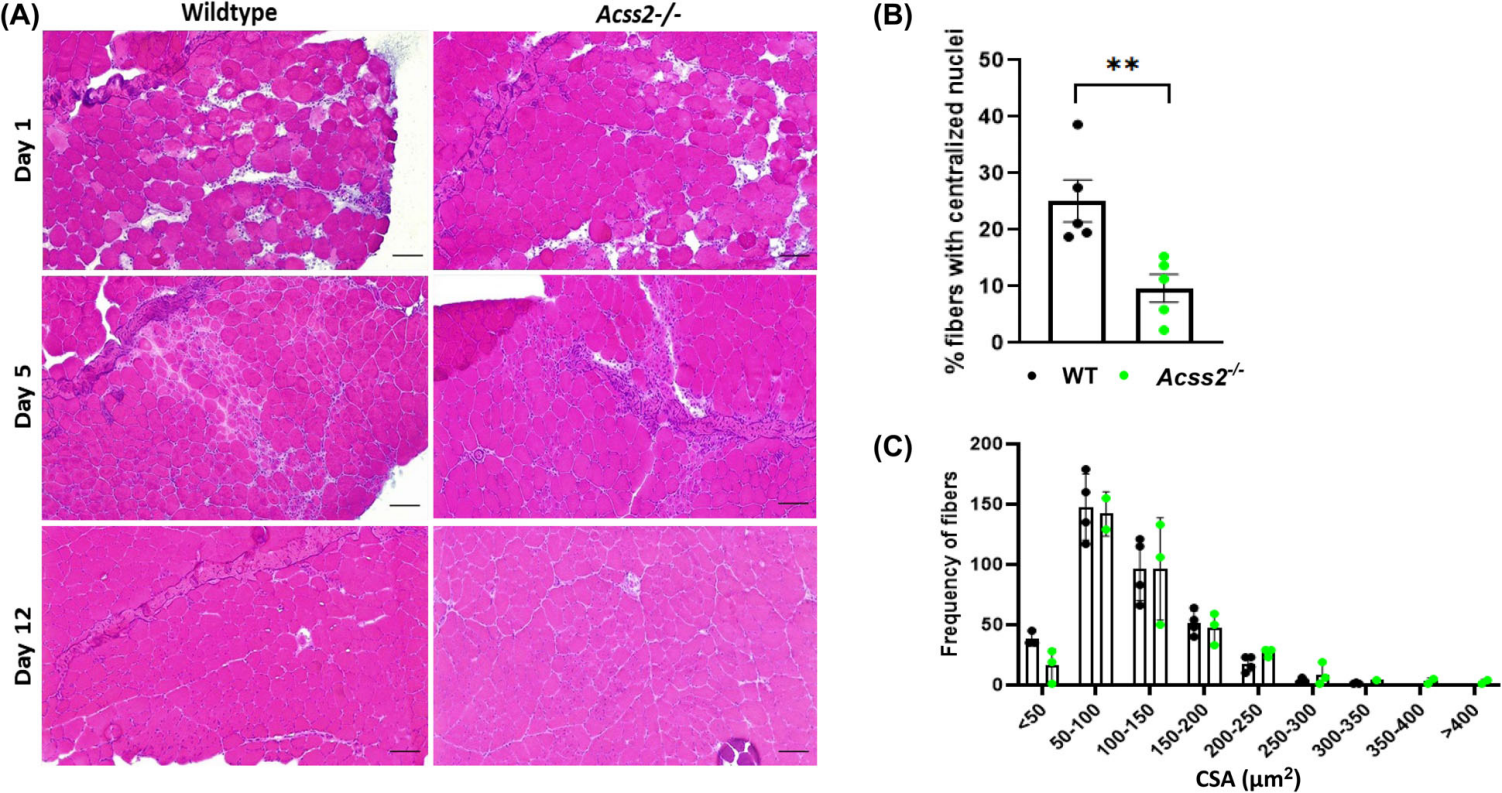
Muscle injury experiment in *Acss2* ^−/−^ mice. (A) H&E staining of *Acss2* ^−/−^ and wild‐type (WT) tibialis anterior (TA) muscles injected with 1.2% barium chloride at 1, 5, and 12 days post‐injury. (B) At day 5, centrally nucleated fibers were less abundant in *Acss2* ^−/−^ mice versus WT. (C) Distribution of the frequencies of various muscle fiber sizes from *n* = 3 mice on day 12 of regeneration was quantified using imagej. An unpaired *t*‐test shows **, *P* < 0.01. The data represent *n* = 5 *Acss2* ^−/−^ and *n* = 5 WT mice. Error bars show standard error of the mean (SEM). Scale bar, 25 μm.

**Fig. 5:**
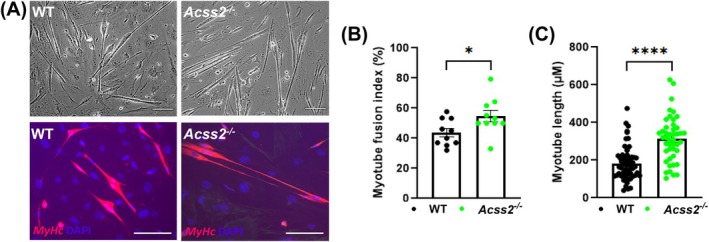
Myoblast differentiation in *Acss2* ^−/−^ mouse muscles. (A) Primary mouse myoblasts (PMM) were isolated from 1‐ to 5‐day‐old *Acss2* ^−/−^ and wild‐type (WT) pups. The cells were differentiated for 4 days, and myotubes were fixed and stained for *MyHc*. Scale bar, 100 μm. *Acss2* ^−/−^ myotubes showed (B) higher fusion rates and (C) increased lengths compared to WT myotubes. The data represent *n* = 2 biological experiments from batches of at least three pups for each genotype. An unpaired *t*‐test shows *, *P* < 0.05 and ****, *P* < 0.0001. Error bars show standard error of the mean (SEM).

Tibialis anterior (TA) muscle specimens were collected from *Acss2* ^−/−^ and WT mice 1, 5, and 12 days after single intramuscular injections of barium chloride. On day 1, there were numerous damaged and necrotic muscle fibers in both the *Acss2* ^−/−^ and WT muscle sections. On day 5, the *Acss2* ^−/−^ muscle specimens displayed fewer regenerating fibers and fewer fibers with centralized nuclei compared to the WT muscle (Fig. 4A,B). However, there were no significant differences in newly regenerated fiber sizes on day 12 (Fig. 4C). Primary mouse myoblasts isolated from *Acss2* ^−/−^ neonatal mice yielded higher myotube fusion indices and longer myotubes than WT myoblasts during differentiation, suggesting the possibility of precocious differentiation in the setting of ACSS2 deficiency (Fig. 5).

### Effects of ACLY inhibition on locomotor function in *Acss2* ^−/−^ mice

**Fig. 6:**
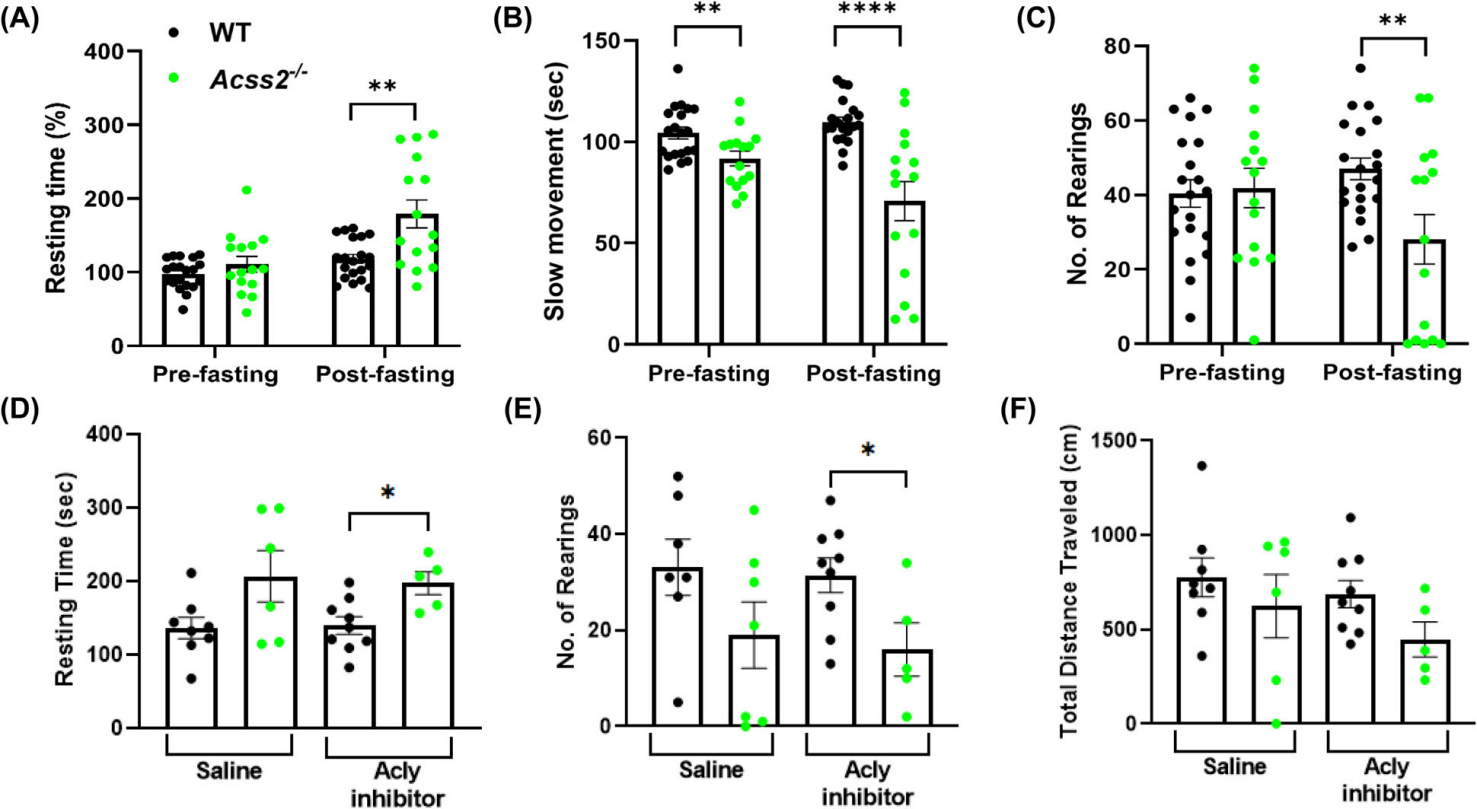
Motor functional activity of *Acss2* ^−/−^ mice. *Acss2* ^−/−^ and wild‐type (WT) mice underwent fasting, exercise, and locomotor analysis using the Actitrak system. (A) The resting times of the *Acss2* ^−/−^ mice were increased post‐fast. (B) Slow movements and (C) the number of rearings were decreased in *Acss2* ^−/−^ mice compared to WT. ACLY inhibition (D) increased resting times and (E) decreased the numbers of rearings of *Acss2* ^−/−^ mice in the post‐fasted condition. (F) There were no significant differences in the total distance traveled of *Acss2* ^−/−^ mice after ACLY inhibition. An unpaired *t*‐test shows *, *P* < 0.05; **, *P* < 0.01; and ****, *P* < 0.0001. The data represent *Acss2* ^−/−^ and WT mice with sexes combined. Error bars show standard error of the mean (SEM).

Locomotor activity analyses measured via Actitrak showed no significant differences between *Acss2* ^−/−^ and WT mice under standard dietary conditions (data not shown). After 16 h of fasting, there were decreases in slow movements and rearings, along with increases in resting time for both male and female *Acss2* ^−/−^ mice compared to WT mice (Fig. 6A–C). In contrast, there were no significant changes in blood glucose, fast movement, and total distance traveled (Fig. S3). ACLY, an enzyme that catalyzes a complementary reaction that also yields acetyl‐CoA, did not show significant protein expression differences in the quadriceps of *Acss2* ^−/−^ mice (Fig. S4). To determine whether ACLY might be compensating for ACSS2 deficiency in skeletal muscles, we administered an ACLY inhibitor to *Acss2* ^−/−^ mice for 7 days followed by 16 h of fasting. Post‐fasting, the *Acss2* ^−/−^ mice treated with the ACLY inhibitor demonstrated delayed locomotor activity including increased resting time, decreased number of rearings, and decreased total distance traveled (Fig. 6D–F) compared to WT mice that were treated with the ACLY inhibitor. There were no significant differences in fast or slow movements, while blood glucose levels were decreased in *Acss2* ^−/−^ mice that were treated with the ACLY inhibitor (Fig. S5).

### Social behavior in *Acss2* ^−/−^ mice

A three chamber behavior assay was performed on the *Acss2* ^−/−^ and WT mice to determine the effect of ACSS2 deficiency on social preference and social recognition. Some social differences were observed in male *Acss2* ^−/−^ mice compared to WT mice, but these findings were not replicated in female *Acss2* ^−/−^ mice compared to WT mice (Fig. S6).

### AcCoA knockdown in *Drosophila*

We generated different tissue‐specific knockdowns of *AcCoA*, the ortholog of *ACSS2* in fruit flies (Table 1 and Fig. S7). The RNAi‐mediated knockdown of *AcCoA* in fat bodies showed potential male lethality, while knockdown in the salivary glands produced smaller‐sized progeny that demonstrated delayed or absent locomotor activity compared to control flies. Some flies also displayed erratic behaviors and movement (Video S1). Curiously, muscle‐specific knockdowns did not yield abnormal phenotypes.

**Table 1:** Tissue‐specific knockdown of *AcCoA* in *Drosophila*.

Targeted tissue/cell type	Viability (*AcCoA* RNAi at 29 °C)
Myoblasts_*Twist‐Gal4*	Viable
Founder muscle cells_*Kirre‐Gal4*	Viable
Muscle_*How‐Gal4*	Viable
Muscle_*Mef2‐Gal4*	Viable
Muscle_*MHC‐Gal4*	Viable
Fat body*_Lsp2‐Gal4*	Male lethal?
Salivary Gland*_323.3‐Gal4*	Viable, some progeny show reduced body size and wing phenotype, most show decreased/absent climbing ability (erratic locomotor activity) Lifespan was not assessed

## Discussion

*ACSS2* is a vital gene involved in catalyzing the conversion of acetate to acetyl‐CoA, which is essential for energy production, fatty acid synthesis, and histone acetylation. Upregulation of ACSS2 has been reported in various cancer tissues. In cancer cells, ACSS2 is upregulated under hypoxic and nutrient deprivation conditions, enabling the cells to overcome unfavorable growth conditions [20]. Silencing of ACSS2 reduces tumor burdens in xenograft models [21]. Pharmacologic inhibition of ACSS2 impairs breast cancer growth [22]. ACSS2 regulates histone acetylation in the nuclei of differentiating neurons in the hippocampus, affecting the spatial memory of adult mice [23]. ACSS2 is also involved in long‐term memory formation by activating autophagy‐related gene expression [24].

Consistent with our findings on ACSS2‐deficient mice, in other contexts ACSS2 positively regulates cell proliferation and migration, favoring cancer cell growth in certain settings [21, 22, 25]. ACSS2 RNA transcripts are abundantly expressed in specific tissues including skeletal muscle and tongue [26]. Given its high rates of physical and metabolic activity, skeletal muscle requires large volumes of energy production. Our findings of intermittent embryonic lethality and growth differences in the *Acss2* ^−/−^ mice, along with the growth and developmental consequences of knocking down the orthologous gene *AcCoA* in fly salivary glands, indicate that the ACSS2 and AcCoA enzymes are critical for growth and development from the embryonic stage onwards in mice and flies. Our findings contrast with those of other studies that found no differences in the phenotype and litter size of *Acss2* ^−/−^ mice [27, 28].

ACSS2 contributes to memory‐related cognitive functions via histone acetylation modulation in the brain [23]. We observed social behavioral differences in male *Acss2* ^−/−^ mice compared to WT mice, but not in female mice. ACSS2‐dependent histone acetylation is a key regulator of cognitive function in mouse models of Alzheimer's disease [29] and in fear memory formation [28]. Our sex‐specific findings are intriguing, particularly given the known male dominance of certain neurodevelopmental disorders, including autism and attention deficit hyperactivity disorder (ADHD) [30, 31].

A novel aspect of our study is the characterization of skeletal muscle consequences of ACSS2 deficiency. ACSS1 is known to be involved in mitochondrial function, while ACSS2 localizes predominantly to the cytosolic and nuclear compartments [32]. ACSS2 plays a role in hepatic lipid storage, deduced from the association of ACSS2 deficiency with decreased expression of lipid and fatty acid synthesis genes in liver [18]. Our data show numerous impacts of ACSS2 deficiency in skeletal muscle, including upregulation of *MuRF‐1* and reduced NADH staining. Reduction of NADH is an indication of mitochondrial dysfunction due to decreased oxidative capacity, which is ultimately involved in ATP‐mediated energy production, glycolysis, and fatty acid oxidation in the target tissues [33, 34]. We also observed abnormal lipid droplet accumulation in *Acss2* ^−/−^ mouse muscles, suggesting impairments of fatty acid oxidation [35]. Another indication of metabolic distress in the setting of ACSS2 deficiency was our finding of a locomotor phenotype under fasting conditions. We thus suspect that defects in lipid metabolism and mitochondrial dysfunction are responsible for the consequences of ACSS2 deficiency in skeletal muscle, rather than altered histone acetylation. Confirmation of the relative contributions of these mechanisms to the skeletal muscle phenotype merits examination in a future study.

ACLY converts glucose‐derived citrate to acetyl co‐A. ACLY‐mediated compensation for the loss of ACSS2 and vice versa are reported previously in several *in vitro* studies [8, 36]. We showed that the locomotor phenotype of *Acss2* ^−/−^ mice was accentuated in the setting of ACLY inhibition, demonstrating that ACLY compensates for some aspects of ACSS2 deficiency *in vivo*. However, given the multiple differences we have seen in *Acss2* ^−/−^ mice, this compensation is not complete for all functions of ACSS2.

In conclusion, we have demonstrated a set of skeletal muscle‐specific features in a mouse model of ACSS2 deficiency, showing that this enzyme is critical to the proper development and function of skeletal muscles. We have also confirmed that ACSS2 and ACLY have some complementary functions in the ACSS2‐deficient mouse and that AcCoA‐deficient *Drosophila* display developmental and locomotor defects. In humans, ACSS2 is an upstream regulator of two major metabolic pathways that lead to cholesterol synthesis and fatty acid synthesis. The functionality of ACSS2 may thus influence the phenotypes of muscular dystrophies caused by defects in mevalonate pathway components, including HMGCS1 [16], HMGCR [12, 13, 37], and GGPS1 [15]. Interestingly, protein interactions between ACSS2 and HMGCS1 have been reported in pancreatic neuroendocrine cells [38]. Future studies that examine interactions between ACSS2 and mevalonate pathway components promise to illuminate mechanisms of muscle‐specific diseases related to these pathways.

## Author contributions

MG and PBK designed the experiments. NMW, GC, MG, and KL performed mouse experiments. MG and NMW performed the primary mouse myoblast experiments. MG, NMW, GC, and KL analyzed the mouse data and created figs. ID performed the *Drosophila* experiments and composed the *Drosophila* section of the manuscript. MG wrote the manuscript, CAP reviewed the data and edited the manuscript, and PBK edited and finalized the manuscript.

## Peer review

The peer review history for this article is available at https://www.webofscience.com/api/gateway/wos/peer‐review/10.1002/1873‐3468.70152.

## Supporting information


**Fig. S1.** Muscle tissue weights measured during necropsy at 11 weeks of age.
**Fig. S2.** Fiber type proportions in muscles from *Acss2* ^−/−^ and wild‐type (WT) mice.
**Fig. S3.** Fasting experiment on *Acss2* ^−/−^ and wild‐type (WT) mice.
**Fig. S4.** ACLY protein levels in muscle.
**Fig. S5.** ACLY inhibitor experiment.
**Fig. S6.** Social behavioral experiments at 10 weeks.
**Fig. S7.** Protein alignment of AcCoA (RefSeq NP_001014599.2) and ACSS2 (RefSeq XP_011527207.1).

**Video S1.** Video of RNAi‐mediated *AcCoA* knockdown flies and control siblings.

## Data Availability

The data that support the findings of this study are available from the corresponding author, PBK, at pkang@umn.edu upon reasonable request.
