# Supplementary material for: ACSS2 involved in acetyl‐CoA synthesis regulates skeletal muscle function
Source: FEBS Lett. 2025 Sep 12;599(19):2817–27. doi: 10.1002/1873-3468.70152 (PMC12519054; doi:10.1002/1873-3468.70152)
Supplement: Supplementary file 1 — Fig. S1. Muscle tissue weights measured during necropsy at 11 weeks of age. Fig. S2. Fiber type proportions in muscles from Acss2 −/− and wild‐type (WT) mice. Fig. S3. Fasting experiment on Acss2 −/− and wild‐type (WT) mice. Fig. S4. ACLY protein levels in muscle. Fig. S5. ACLY inhibitor experiment. Fig. S6. Social behavioral experiments at 10 weeks. Fig. S7. Protein alignment of AcCoA (RefSeq NP_001014599.2) and ACSS2 (RefSeq XP_011527207.1). [file FEB2-599-2817-s001.pdf]

ACSS2 involved in acetyl-CoA synthesis regulates skeletal muscle function

Supplemental Material

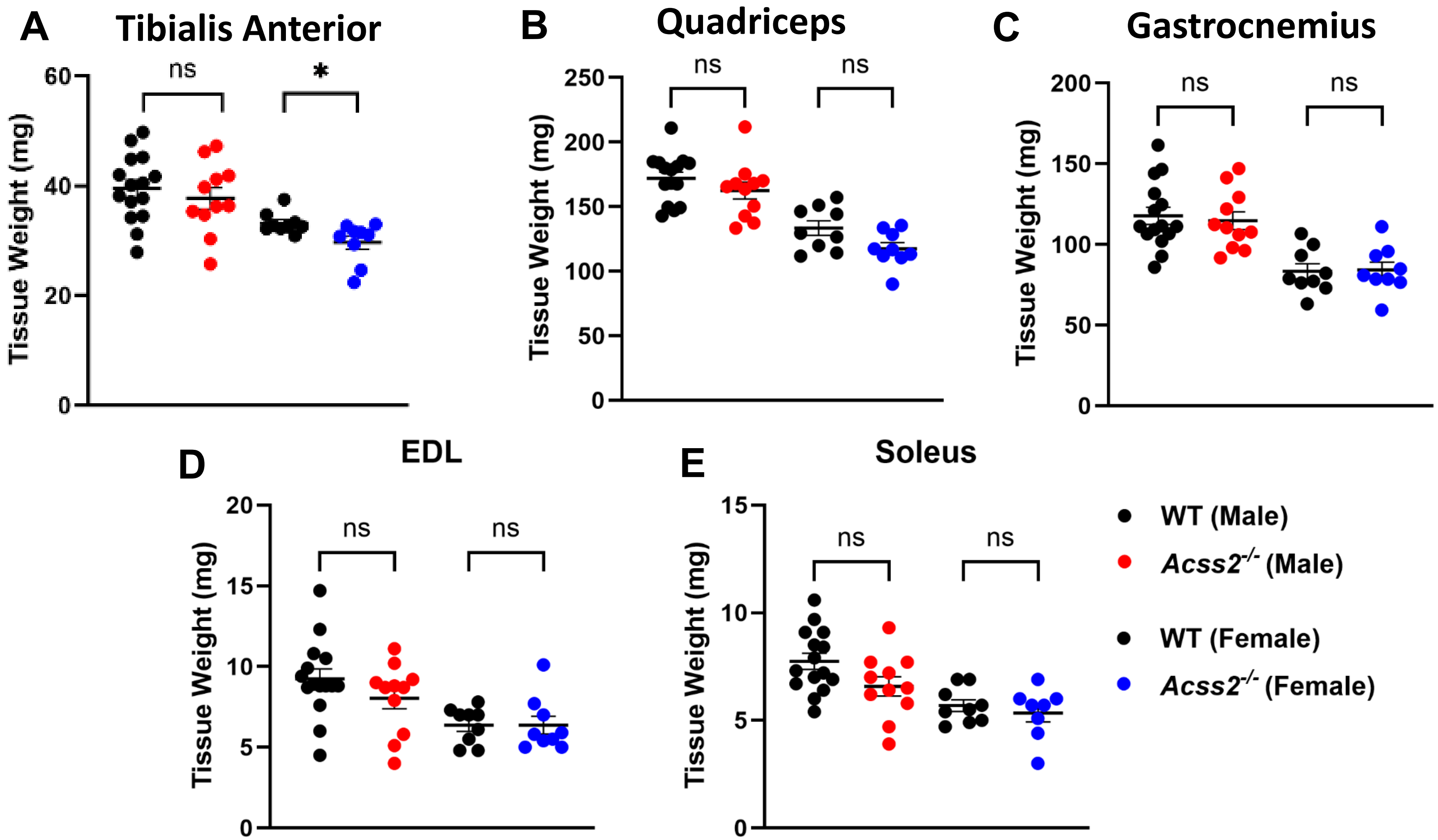

Figure S1. Muscle tissue weights measured during necropsy at 11 weeks of age. (A) Female *Acss2*$^{-/-}$ tibialis anterior (TA) muscles had slightly reduced weights compared to female wild type (WT) TA muscles. The (B) quadriceps, (C) gastrocnemius, (D) EDL, and (E) soleus muscles showed no significant differences between genotypes. An unpaired t-test shows *, $p<0.05$. The data represent n=11 (male *Acss2*$^{-/-}$), n=15 (male WT), n=9 (female *Acss2*$^{-/-}$), and n=9 (female WT). Error bars show standard error of the mean (SEM).

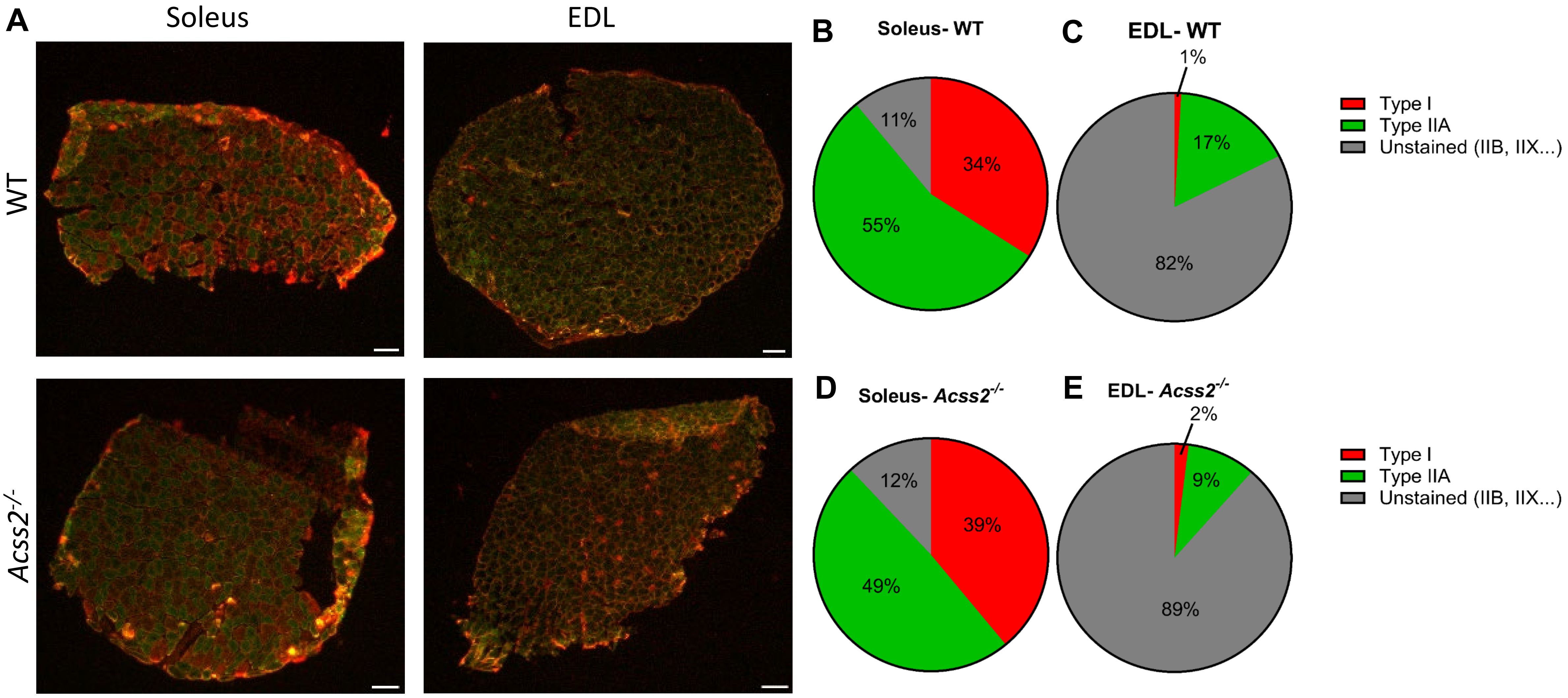

Figure S2. Fiber type proportions in muscles from *Acss2*$^{-/-}$ and wild type (WT) mice. (A) Representative images of soleus and EDL muscles from *Acss2*$^{-/-}$ and WT mice. Scale bar, 100*μm.* Muscle fiber types were stained using MHC-I and MHC-IIA antibodies staining slow and fast fibers, respectively. (B-E) Fiber type abundance was quantified using ImageJ. No significance differences were found. Results show n=6 biological replicates.

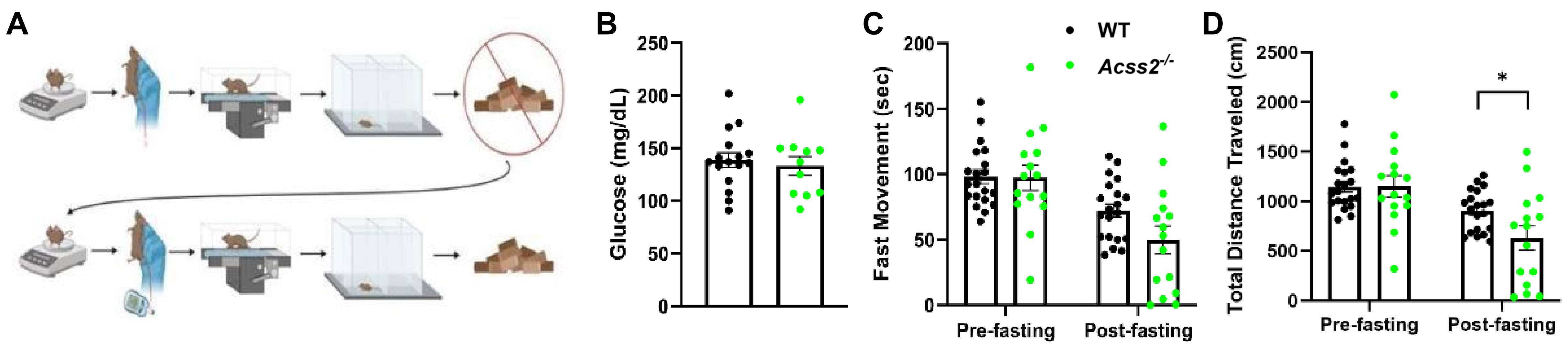

Figure S3. Fasting experiment on *Acss2*$^{-/-}$ and wild type (WT) mice. (A) Fasting experiment workflow is shown. Diagram was generated using BioRender under a license permitting their use in publications. *Acss2*$^{-/-}$ and wild type (WT) mice underwent fasting, exercise and locomotor analysis using Actitrak. Blood glucose levels were measured from the tail vein of *Acss2*$^{-/-}$ and WT male and female mice before and after 16 hours of fasting. (B) There were no significant differences in glucose levels between *Acss2*$^{-/-}$ and WT mice post-fasting. Locomotor activity was measured before and after fasting after exercising at a 15° incline for 5 minutes at 5m/min and then 10 minutes at 10m/min. (C) There were no significant differences in fast movements. (D) A slight difference was noted in total distance traveled between *Acss2*$^{-/-}$ and WT mice of both sexes in the post-fasted condition. The data represent n=5 (male *Acss2*$^{-/-}$), n=10 (male WT), n=10 (female *Acss2*$^{-/-}$), and n=10 (female WT). Outliers were removed from male and female WT groups. An unpaired t-test shows *, $p<0.05$. Error bars show standard error of the mean (SEM).

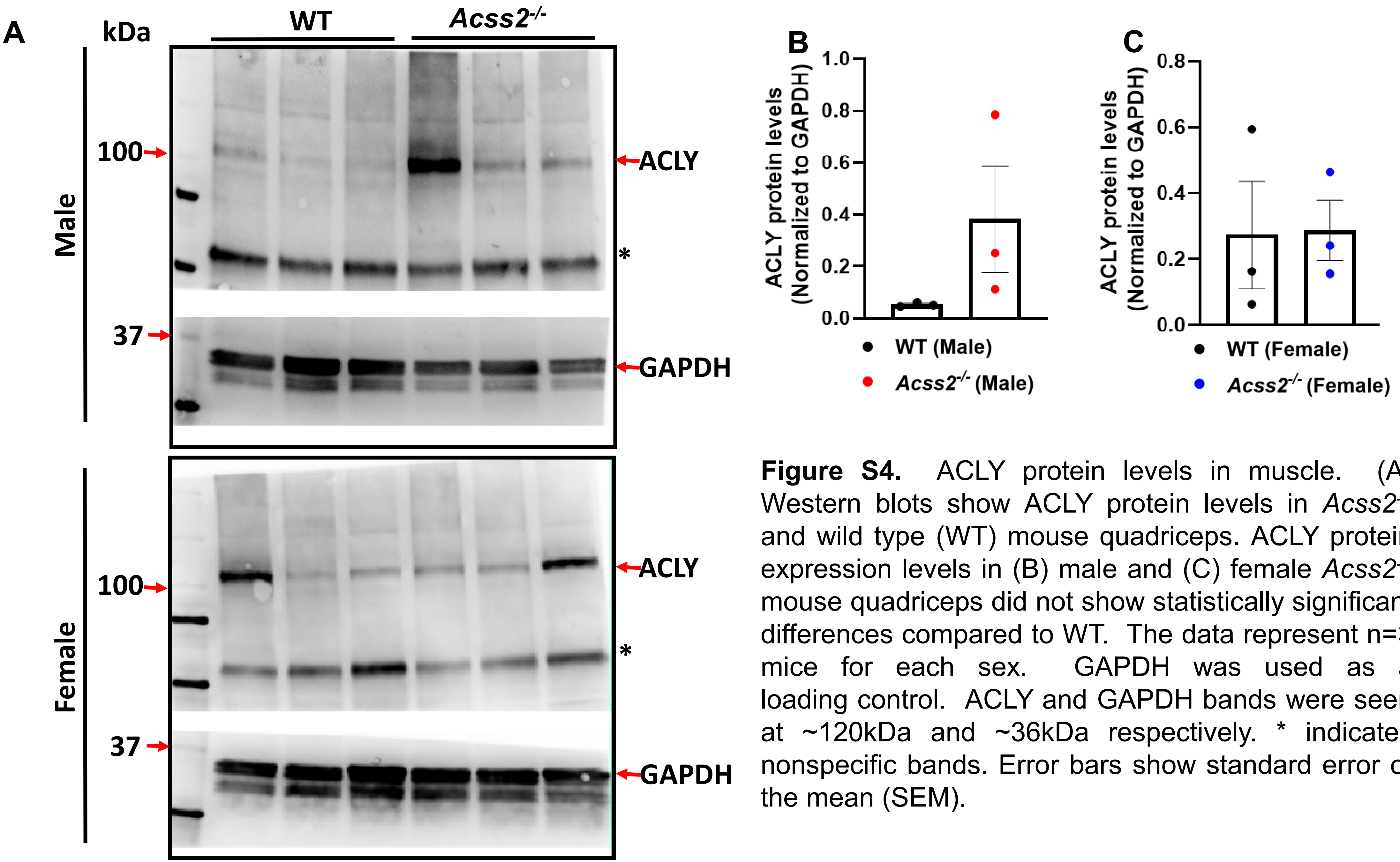

Figure S4. ACLY protein levels in muscle. (A) Western blots show ACLY protein levels in *Acss2*$^{-/-}$ and wild type (WT) mouse quadriceps. ACLY protein expression levels in (B) male and (C) female *Acss2*$^{-/-}$ mouse quadriceps did not show statistically significant differences compared to WT. The data represent n=3 mice for each sex. GAPDH was used as a loading control. ACLY and GAPDH bands were seen at ~120kDa and ~36kDa respectively. * indicates nonspecific bands. Error bars show standard error of the mean (SEM).

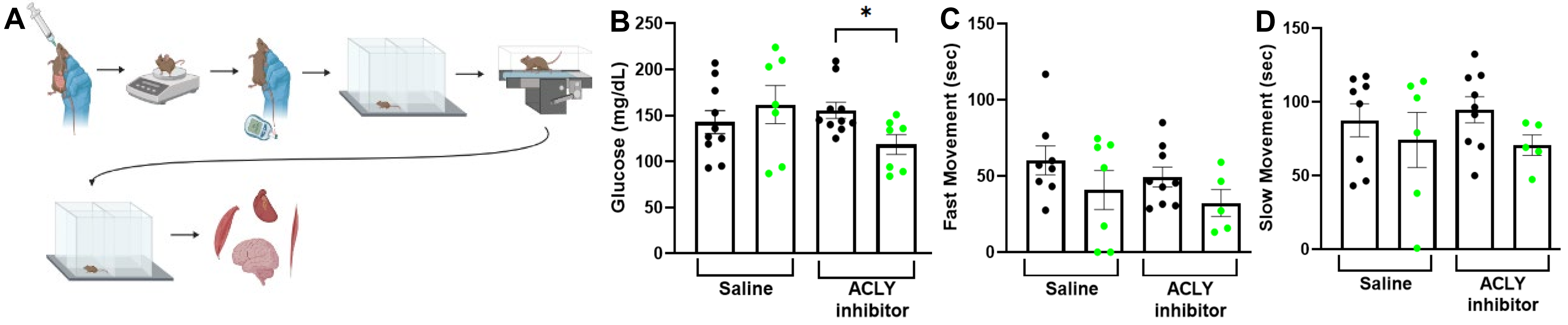

Figure S5. ACLY inhibitor experiment. (A) ACLY inhibitor experiment workflow is shown. Diagram was generated using BioRender under a license permitting their use in publications. *Acss2*$^{-/-}$ and wild type (WT) mice underwent exercise and locomotor analysis using Actitrak. Blood glucose levels were measured from the tail vein of *Acss2*$^{-/-}$ and WT male and female mice after 7 days of ACLY inhibitor or oral saline administration. (B) There was a mild decrease in glucose levels in the *Acss2*$^{-/-}$ ACLY inhibitor group. The mice underwent exercise and locomotor analysis using Actitrak. There were no significant differences in (C) fast or (D) slow movements between *Acss2*$^{-/-}$ and WT mice in the ACLY inhibitor and saline groups. An unpaired t-test shows *, $p<0.05$. Error bars show standard error of the mean (SEM).

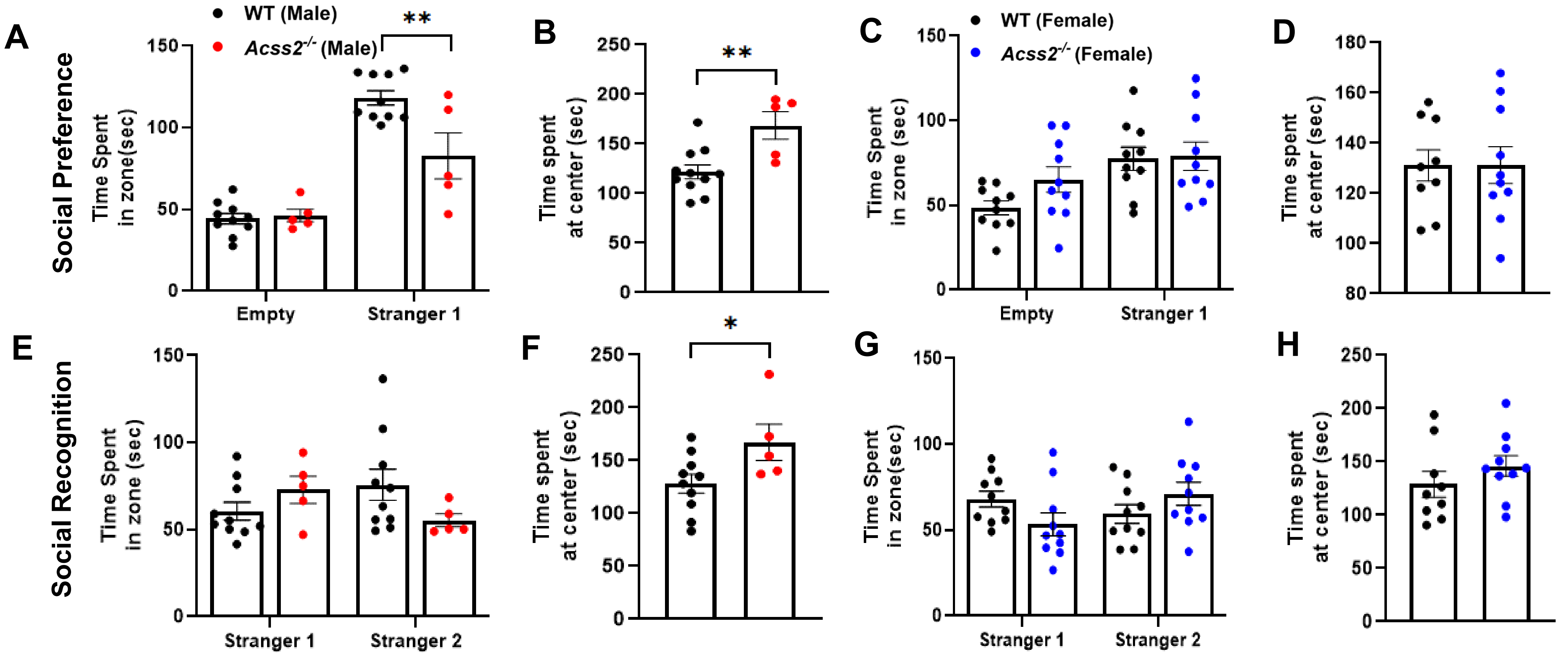

Figure S6. Social behavioral experiments at 10 weeks. (A) *Acss2*$^{-/-}$ male mice interacted less with stranger mice compared to wild type WT mice. (B) *Acss2*$^{-/-}$ male mice spent more time in the center, indicating less exploratory interest. (C and D) In contrast, *Acss2*$^{-/-}$ female mice did not display differences in social preferences compared to WT mice. (E and F) There was a subtle difference between *Acss2*$^{-/-}$ and WT male mice in time spent in the center. (G and H) *Acss2*$^{-/-}$ female mice did not display differences in social recognition compared to WT mice. An unpaired t-test shows *, $p < 0.05$; **, $p < 0.01$. The data represent n=5 (male *Acss2*$^{-/-}$), n=10 (male WT), n=10 (female *Acss2*$^{-/-}$), and n=10 (female WT). Error bars show standard error of the mean (SEM). Outliers have been removed from the male WT group.

```
  Fly      8 YDPNPAISQNAYISSFEEYQKFYQESLDNPAEFWSRVAKQFHWETPADQDKFLKYNFNISKGPIS 72
             :.|.|.:|::|::.|.:.|::.::.|::.|.|||..:||:|:|:||. ...||:|||:::||.|.
Human     29 WSPPPEVSRSAHVPSLQRYRELHRRSVEEPREFWGDIAKEFYWKTPC-PGPFLRYNFDVTKGKIF 92

  Fly     73 IKWMEGASTNLCYNLLDRNV-RNGLGDQIAYYW-------------EGNHPDDYSRGLTYRKLLE 123
             |:||:||:||:|||:||||| ...|||::|:||             |||.|.:.:: :||.:||.
Human     93 IEWMKGATTNICYNVLDRNVHEKKLGDKVAFYWSTSGNSSYRYTCREGNEPGETTQ-ITYHQLLV 156

  Fly    124 EVCRFANVLKDHGIRKGDRVSIYMPMILELPIAMLACARIGAVHSIVFAGFSPDSLAERMFDCKA 188
             :||:|:|||:..||:|||||:||||||.||.:||||||||||:|||||||||.:||.||:.|...
Human    157 QVCQFSNVLRKQGIQKGDRVAIYMPMIPELVVAMLACARIGALHSIVFAGFSSESLCERILDSSC 221

  Fly    189 KLLITADGAWRGEKPLYLKALCDTALEKVEEMGHSVEKCIVVSHLKRV----------TP----- 238
             .||||.|..:||||.:.||.|.|.||:|.:|.|..|..||||.||.|.          :|
Human    222 SLLITTDAFYRGEKLVNLKELADEALQKCQEKGFPVRCCIVVKHLGRAELGMGDSTSQSPPIKRS 286

  Fly    239 CQPD----------HVEEEIPWTDDRDYWWHEEMEDKEPACYPEWMDAEDPLFMLYTSGSTGKPK 293
             | ||          .|:.:|.|....|.||||.|::....|.|||.|||||||:|||||||||||
Human    287 C-PDVQGKLKEKSKRVQPQISWNQGIDLWWHELMQEAGDECEPEWCDAEDPLFILYTSGSTGKPK 350

  Fly    294 GVLHTTAGYLLYAATTFKIVFDYKPGDIYWCTGDVGWITGHTYVVYGPLANGATSVIFEGTPFFP 358
             ||:||..||:||.|||||.|||:...|::|||.|:||||||:||.|||||||||||:|||.|.:|
Human    351 GVVHTVGGYMLYVATTFKYVFDFHAEDVFWCTADIGWITGHSYVTYGPLANGATSVLFEGIPTYP 415

  Fly    359 GNDRYWSVIDKYKVTQFYTAPTAIRALMKFGEGPVLKHNLSGLKVLGSVGEPINPEAWLWYYKYI 423
             ..:|.||::||||||:|||||||||.|||||:.||.||:.:.|:|||:|||||||||||||::.:
Human    416 DVNRLWSIVDKYKVTKFYTAPTAIRLLMKFGDEPVTKHSRASLQVLGTVGEPINPEAWLWYHRVV 480

  Fly    424 GKEQCSIVDTFWQTETGGHVITPLPGATPMKPGSASFPFFGVKPTLLDECGIEIKGEGEGYL--- 485
             |.::|.|||||||||||||::||||||||||||||:||||||.|.:|:|.|.|::||.||||
Human    481 GAQRCPIVDTFWQTETGGHMLTPLPGATPMKPGSATFPFFGVAPAILNESGEELEGEAEGYLLLR 545

  Fly    486 -------VFSQPWPGMMRTLYNNHERFEDTYFSKFPGYYCTGDGARRDADGYLWITGRVDDMLNV 543
                    ||.|||||:|||:|.||||||.|||.||||||.||||.:||.|||.|||||:||||||
Human    546 TETSWLEVFKQPWPGIMRTVYGNHERFETTYFKKFPGYYVTGDGCQRDQDGYYWITGRIDDMLNV 610

  Fly    544 SGHLMSTAEVESVLTEHPRVAESAVVSRPHPVKGECLYCFITPNENEVFDQKLISDLKKMVRERI 608
             ||||:|||||||.|.||..|||:|||..||||||||||||:|..:...|..||..:|||.:||:|
Human    611 SGHLLSTAEVESALVEHEAVAEAAVVGHPHPVKGECLYCFVTLCDGHTFSPKLTEELKKQIREKI 675

  Fly    609 GPFAMPDVIQNAPGLPKTRSGKIMRRVLRKIAVNDRNVGDTSTLADEQIVEQLFANR 665
             ||.|.||.||||||||||||||||||||||||.||.::||.||:||..::..||::|
Human    676 GPIATPDYIQNAPGLPKTRSGKIMRRVLRKIAQNDHDLGDMSTVADPSVISHLFSHR 732
```

Figure S7. Protein alignment of AcCoA (RefSeq NP_001014599.2) and ACSS2 (RefSeq XP_011527207.1). There is 74% similarity between the fly and human orthologs. Obtained from FlyBase (https://flybase.org).

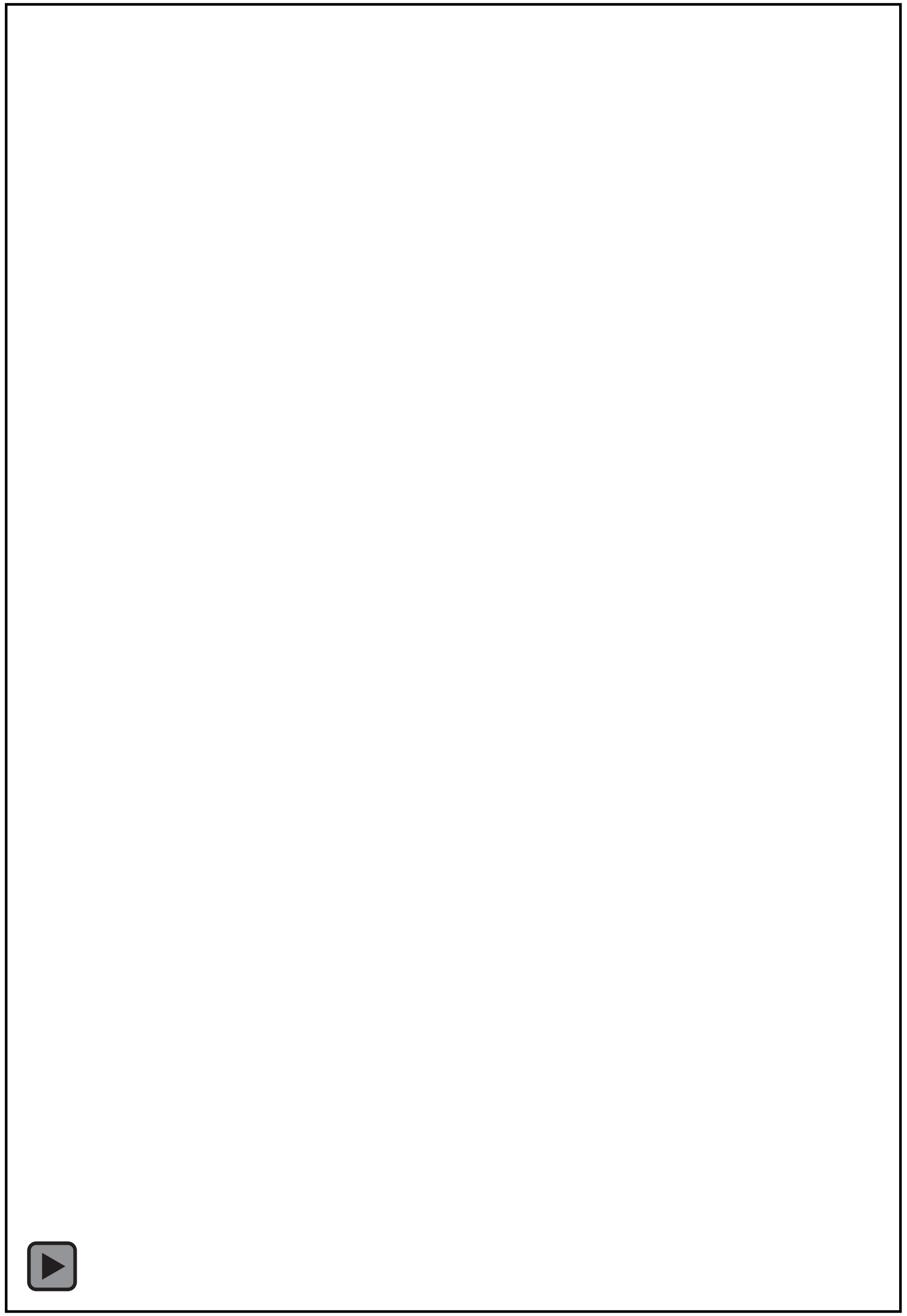

Video S1. Video of RNAi mediated *AcCoA* knockdown flies and control siblings. The flies were generated at 29°C using Indiana University Bloomington Drosophila stock Center (BDSC) stock #41917, genotype: *y[1]sc[*] v[1] sev[21]; P{y[+t7.7] v[+t1.8]=TRiP.HMS02314}attP2*) and the salivary gland Gal4 driver BDSC stock # 5398, genotype w[*]; P{w[+mW.hs]=GawB}332.3. In the corresponding video, the *AcCoA* knockdown flies (right) show delayed/ erratic locomotor activity compared to control flies (left).
